# Patient-Physician Messaging by Race, Ethnicity, Insurance Type, and Preferred Language

**DOI:** 10.1001/jamanetworkopen.2025.34549

**Published:** 2025-10-07

**Authors:** Lisa S. Rotenstein, Brianna Hardy, Mitchell Tang, Bryan Steitz, Robert W. Turer, Emily Alsentzer, Michael L. Barnett

**Affiliations:** 1 Brigham and Women’s Hospital, Boston, Massachusetts; 2 University of California at San Francisco; 3 Harvard Business School, Boston, Massachusetts; 4 Vanderbilt University Medical Center, Nashville, Tennessee; 5 University of Texas Southwestern Medical Center, Dallas; 6 Harvard T.H. Chan School of Public Health, Boston, Massachusetts

## Abstract

**Importance:**

Asynchronous patient-portal messaging has emerged as a central component of patient-physician communication, yet disparities in response patterns remain underexplored.

**Objective:**

To examine variations in asynchronous messaging responses in primary care by race and ethnicity, insurance type, and primary language.

**Design, Setting, and Participants:**

This cross-sectional study of messaging data included 795 170 adult patients receiving primary care at Mass General Brigham in 2021, 341 836 of whom sent an asynchronous patient portal message. Data were analyzed from April 17, 2023, to July 29, 2025.

**Exposures:**

Race and ethnicity, insurance status, and preferred language.

**Main Outcomes and Measures:**

Receipt of a response within 1 or 3 business days and time to first response from care team. Multivariable regression models adjusted for patient demographics, clinic, and time fixed effects.

**Results:**

The analytic sample consisted of 341 836 patients, who sent a total of 3 525 905 messages comprising 1 270 662 message threads to 1113 physicians. Among these patients (mean [SD] age, 52.2 [17.3] years), 18 442 were Asian (5.4%), 14 089 were Black or African American (4.1%), 9979 were Hispanic or Latino (2.9%), 285 919 were White (83.6%), 232 661 had commercial insurance (68.1%), and 332 004 primarily spoke English (97.1%). Characteristics of patient-threads that received a response from any care team member within 1 business day were as follows: 65.7% of threads from Black participants (32 165 of 48 983), 63.9% of threads from Hispanic or Latino (21 732 of 34 014), 68.5% of threads from White participants (743 161 of 1 085 517), 70.0% of threads from patients with commercial insurance (529 803 of 756 923), 60.9% of threads from patients with dual-eligibility (40 846 of 67 045), 68.4% of threads from patients preferring English (847 489 of 1 239 768), and 58.0% of threads from patients preferring Spanish (6898 of 11 903). Similar disparities were found in the response rate from primary care physicians. After adjustment, compared with White patients, the rate of response from any care team member within 1 day was lower for Black patients (1.1 [95% CI, 0.2-2.0] percentage points; *P* = .01) and Hispanic patients (1.1 [95% CI, 0.3-1.9] percentage points; *P* = .01). Compared with patients with commerical insurance, the response rate within 1 day was lower for dual-eligible patients (4.9 [95% CI, 4.2-5.5] percentage points; *P* < .001), and compared with patients who preferred English, the response rate within 1 day was lower for patients who preferred Spanish (4.1 [95% CI, 2.5-5.7] percentage points; *P* < .001). Adjustment for clinic and time fixed effects accounted for most of the observed disparities in response rates and timing.

**Conclusions and Relevance:**

In this cross-sectional study of primary care patients in a single health system, there were significant disparities in the responsiveness of primary care teams to asynchronous patient-portal messages by race and ethnicity, insurance type, and language. These were partially attributable to slower response times at practices that treat underserved patients.

## Key Points

**Question:**

Are there differences in the patterns of care teams' responses to asynchronous patient portal messages by patient demographic characteristics?

**Findings:**

In this cross-sectional study of 341 836 patients who sent asynchronous patient portal messages to their primary care physician, the prevalence of receiving a response from any care team member within 1 business day was lower for Black and Hispanic patients compared with White patients, dual-eligible patients compared with commerically insured patients, and Spanish-preferring compared with English-preferring patients.

**Meaning:**

These findings suggest that there were significant differences in the responsiveness of primary care teams to asynchronous patient-portal messages by race and ethnicity, insurance type, and language.

## Teaser

This cross-sectional study examines variations in messaging responses from primary care teams by race and ethnicity, insurance type, and primary language.

## Introduction

Electronic health record (EHR) based asynchronous messaging, also referred to as patient portal messaging, has become a ubiquitous feature of patient-physician communication. In the US, use of patient portal messaging has become particularly prevalent since the advent of the Health Information Technology for Economic and Clinical Health Act, which mandated communication with patients via electronic patient portals.^1^ Use of asynchronous patient portals has also been described outside of the US,^2,3^ although less time is spent by physicians on this activity in non-US settings.^4^ The centrality of patient portal messaging to health care delivery has only increased since 2020, when patient portal message quantity more than doubled as organizations encouraged asynchronous communication in lieu of in-person visits during the COVID-19 pandemic.^5^ Even after social distancing restrictions lifted, patients and their care teams continue to use asynchronous patient portal-based messaging at higher rates than prior to the pandemic.

The rising importance of patient portal messaging raises the stakes for any disparities among patients being offered and accessing patient portals. Patients message their care teams for a variety of reasons, ranging from discussing new or existing symptoms, corresponding regarding management of chronic diseases, asking questions about medication management, and discussing visit logistics or paperwork, among many potential examples. Similar to health disparities in other domains, patient portal access differs greatly by patient race and ethnicity. For example, in a national study, Black and Hispanic individuals were both offered and accessed patient portals at significantly lower rates than White individuals.^6^ In a large Midwestern health system, as compared with White patients with hypertension, non-Hispanic Black and Hispanic patients with hypertension had lower odds of any access to a patient portal, access around the time of a primary care physician (PCP) visit, and messaging via the portal.^7^ Disparities by language have also been documented; in a large, urban safety-net health care system, preferring English as one’s primary language and being US-born were associated with greater odds of active portal use.^8^

There is less evidence on how patients’ interactions with care team members may differ once they are using asynchronous patient portal-based messaging. Given the proliferation of electronic, asynchronous communication between patients and their care teams, many institutions have developed standards for response times to electronic patient messages, commonly with an expected response time of 3 business days.^9,10^ Slower, less responsive, or more negative interactions with physicians on patient portals could systematically discourage certain patient groups from engaging in this form of communication. For example, among patients in a large safety net health care system, those from minoritized racial and ethnic groups were less likely to receive replies to patient portal messages from attending physicians and more likely to receive responses from registered nurses.^11^ Understanding these interactions is critical given evidence of differential experiences of care receipt by patients who are not White, do not prefer to speak English as their primary language, and do not have commercial insurance across the US health care system.^12,13,14,15,16^

In this study, we aimed to characterize how response patterns to patients’ asynchronous portal messages varied across a large, integrated primary care network by race and ethnicity, insurance type, and primary language. Specifically, we sought to determine whether patient characteristics were associated with differences in the proportion of patients receiving a response to a message from a care team member and the distribution of response times to asynchronous patient messages.

## Methods

This cross-sectional study was deemed exempt by the Mass General Brigham (MGB) institutional review board (IRB), with a waiver of consent provided by the IRB because of the retrospective nature of the study. This study followed the Strengthening the Reporting of Observational Studies in Epidemiology (STROBE) reporting guideline.^19^

### Study Population and Data Sources

Data were derived from the MGB Electronic Data Warehouse, a longitudinally maintained database that includes both structured and unstructured data from the Clarity database underlying the Epic EHR system. The MGB system includes 2 large urban academic medical centers (Brigham and Women’s Hospital and Massachusetts General Hospital), 7 community hospitals, and hundreds of outpatient practices across eastern Massachusetts.

Our cross-sectional study sample included messages to and from adult patients who had a primary care encounter (office-based or virtual) in the MGB health care system in 2021. Primary care encounters were defined as in-person or telemedicine visits among adults aged 18 years and older with an attending physician, fellow, or resident that took place in the following settings: adolescent medicine, family medicine, internal medicine, gerontology, urgent care, or primary care. A small number of patients were listed with missing sex and excluded from our analyses. The eFigure in Supplement 1 displays study inclusion and exclusion criteria.

### Capturing Messages and Respondents

The primary unit of analysis in this study was a message thread that included a patient-initiated electronic patient medical advice request message (referred to as a message) and responses from clinical team members, if any. Each patient message thread was addressed to a single recipient who was typically the primary care clinician for the patient within the clinic (eg, To the Office of Jane Smith, MD). In MGB practices, these messages are typically first screened and triaged by clinic staff, including nurses, medical assistants, and administrative staff (collectively referred to as staff), who may respond directly to the patient or forward the message to the primary care clinician to respond. When the addressed recipient of the message matched the message responder, we flagged the message as having a PCP responding to the message. If not, it was flagged that a staff member responded to the message. The group of all potential responders, staff, or PCP was referred to as the care team. The timing of care team responses to the initial patient message in the thread was used to generate study outcomes.

### Study Outcomes

The primary outcome of the study was the care team’s response time to the first patient message of a thread. This calculation was based on the time difference in minutes between the first patient message and the first care team response within a thread, regardless of whether the responder was their PCP or a staff member. To minimize the influence of outliers (eg, messages that received a response after weeks or months), care team response time was top-coded at the 99th percentile level (ie, all response times of more than 17 065 minutes were replaced by the value of 17 065). We also defined 2 binary outcomes indicating whether the patient message received a response within 1 or 3 business days. We also calculated PCP response time to the first patient message within the thread, estimated with the same approach but focusing on threads with a PCP response. Additional outcomes included a binary outcome indicating whether the patient message had ever received a response from either a staff member or their PCP, and whether the patient message had ever received a response from a PCP.

### Patient and Physician Variables

For all patients included in the analysis, we extracted race and ethnicity, primary insurance type, age, preferred language, and Elixhauser score using outpatient diagnosis codes^17^ variables as represented in the EHR. Patients identify their race, ethnicity, and preferred language from a list of available categories during registration with the MGB system, either directly or with the assistance of registration staff. Race and ethnicity were combined into 1 categorical variable such that if a patient’s race was listed as other and their ethnicity listed as Hispanic or Latino, their race and ethnicity was defined as Hispanic or Latino. Race listed as declined or unavailable was combined into 1 race and ethnicity category. Due to the small number of patients, American Indian or Alaska Native and Native American or Other Pacific Islander were combined into 1 category. We also extracted information about each patient’s zip code during the study period.

Insurance type was defined as the most recent insurance coverage the patient had as of January 1, 2021, for consistency across patients. Age was calculated by subtracting January 1, 2021, from the patient’s birthdate and then dividing it into 7 age categories: 18 to 27 years, 28 to 37 years, 38 to 47 years, 48 to 57 years, 58 to 67 years, 68 to 79 years, and 80 years or older. Primary language was divided into 3 categories: English, Spanish, and all other primary languages (or missing language). All active diagnoses listed in the patient’s problem list as of January 1, 2021, were used for calculating an Elixhauser comorbidity score using the van Walraven weighting.^17,18^ The individual comorbidity flags used in the Elixhauser calculation were additionally included in our models.

The time a message was sent was another important covariate, which would effect when it is responded to (eg, sent on a weekend or in the evening). We used 2 sets of time indicators for modeling purposes: day of the year (365 separate indicators) and hour of the day (24 indicators). For each PCP in the sample, we captured their sex and the primary care clinic where they practice, as captured in the messaging database.

### Statistical Analyses

#### Primary Analysis

We first described the characteristics of patients with primary care encounters in 2021 who additionally sent messages to their primary care teams. We then quantified the proportion of patients who received a first response to their message from any team member (staff or PCP) within 1 or 3 business days and the proportion of patients who received any response to their message from a PCP. We separately described continuous response times to a first patient message from any team member and from a PCP. We then stratified these outcomes by patients’ race and ethnicity, insurance status, and primary language. We subsequently built multivariable linear regression models with the outcomes described previously. These were specified as linear probability models for binary outcomes (eg, response within 1 business day) or as continuous linear outcomes for the outcome of median response time. All models were adjusted for patients’ race and ethnicity, insurance type, age category, sex, primary language, Elixhauser score, and PCP sex. We examined models with different combinations of fixed effects. The first set of adjusted models included fixed effects for day of the year and hour of the day of the first patient message (subsequently referred to as time fixed effects) to address potential differences in the timing of messages sent by different patient groups. This first set of models also included fixed effects for the patient’s zip code of residence. This served as a proxy for socioeconomic status. A second set of adjusted models additionally included fixed effects for each individual clinic. This second set of models facilitates assessment of the contribution of individual primary care clinics to response times. Standard errors were clustered by physician.

Finally, given that response times could be influenced by the content of messages themselves, which could vary by patient group, we adjusted for message content in a final set of adjusted models, which also included the covariates and fixed effects described above. To capture message content, each message thread was assigned to 1 of 20 message categories (number determined by consensus labeling of categories) based on the text of the initiating message. Categories were determined using a latent Dirichlet allocation (LDA) topic model, which performed an unsupervised clustering of the messages based on their word frequencies. We then used the LDA-generated message categories as fixed-effect controls in the primary statistical analyses above to determine if message content was associated with message response within 1 business day (eMethods in Supplement 1). Data analysis occurred from April 17, 2023, to July 29, 2025. Statistical significance was set at α < .05, and R version 4.5.0 (R Project for Statistical Computing) was used for analysis.

#### Sensitivity Analyses

Given that a clinical team might deem a telephone or in-person encounter more appropriate than a message response, we conducted sensitivity analyses to evaluate the percentage of messages for which patients received any message reply or had a telephone or in-person encounter with the same clinic within 1 or 3 business days. Additionally, as an alternative to linear probability models, we specified logistic regression models for our binary outcomes of any care team response in 1 business day or any care team response in 3 business days.

## Results

The study sample consisted of 795 170 patients who had a primary care encounter at MGB in 2021 (Table 1). Of these, 341 836 patients sent at least 1 message during the study period. These patients sent a total of 3 525 905 messages comprising 1 270 662 message threads to 1113 physicians (eFigure in Supplement 1). Among the sample who sent a message to their PCP, 211 496 were female (61.9%), and 208 389 were patients aged 48 years or older (60.9%). English was the primary language preferred by 332 004 patients (97.1%) in the sample, 3824 patients (1.1%) preferred Spanish as their primary language, and 6008 (1.8%) preferred another language or did not have their primary language specified. Among patients who sent a message, 18 442 were Asian (5.4%), 14 089 were Black or African American (4.1%), 9979 were Hispanic or Latino (2.9%), 285 919 were White (83.6%), and 4036 identified as other (1.2%). Most patients who sent a message were commercially insured (232 661 [68.1%]), with 72 023 covered by Medicare (21.1%), 24 063 by Medicaid (7.0%), 10 887 with dual eligibility (3.2%), and 852 with other insurance (0.2%). Characteristics of patients counted by threads and messages are shown in eTable 1 in Supplement 1.

**Table 1.** Characteristics of Patients in Sample

Characteristics	Patients, No. (%)		Percentage of patients with an encounter that sent a message, %
	Patients with an encounter 2021	All patients with a message	
Total	795 170 (100.0)	341 836 (100.0)	43.0
Patient race			
Asian	41 518 (5.2)	18 442 (5.4)	44.4
Black or African American	39 770 (5.0)	14 089 (4.1)	35.4
Declined	10 980 (1.4)	8038 (2.4)	73.2
Hispanic	30 163 (3.8)	9979 (2.9)	33.1
Missing or unavailable	16 279 (2.1)	444 (0.1)	2.7
Native American or Pacific Islander	2050 (0.3)	889 (0.3)	43.4
White	644 721 (81.1)	285 919 (83.6)	44.4
Other^a^	9689 (1.2)	4036 (1.2)	41.7
Patient insurance			
Commercial	511 917 (64.4)	232 661 (68.1)	45.5
Medicaid	65 500 (8.2)	24 063 (7.0)	36.7
Medicare	169 182 (21.3)	72 023 (21.1)	42.6
Dual	38 004 (4.8)	10 887 (3.2)	28.7
Other^b^	2920 (0.4)	852 (0.2)	29.2
Missing or unavailable	7647 (1.0)	1350 (0.4)	17.7
Age, y			
18-27	78 472 (9.9)	32 686 (9.6)	41.7
28-37	109 091 (13.7)	51 094 (14.9)	46.8
38-47	112 126 (14.1)	49 667 (14.5)	44.3
48-57	148 411 (18.7)	64 832 (19.0)	43.7
58-67	159 756 (20.1)	71 218 (20.8)	44.6
68-79	134 738 (16.9)	57 151 (16.7)	42.4
≥80	52 576 (6.6)	15 188 (4.4)	28.9
Sex			
Female	459 016 (57.7)	211 496 (61.9)	46.1
Male	336 154 (42.3)	130 340 (38.1)	38.8
Primary language			
English	747 114 (94.0)	332 004 (97.1)	44.4
Spanish	22 426 (2.8)	3824 (1.1)	17.1
Other^c^	17 442 (2.2%)	4455 (1.3)	25.5
Missing or unavailable	8188 (1.0%)	1553 (0.5)	19.0
Elixhauser score, mean (SD)^d^	2.02 (−6.37)	1.94 (−6.23)	NA

Abbreviation: NA, not applicable.

^a^ The patient did not identify their race or ethnicity among those listed.

^b^ That the patient had insurance listed but it was not 1 of the 4 named categories.

^c^ That the patient’s primary language was not English or Spanish or was missing.

^d^ The van Walraven weighted Elixhauser score^17^ was used which calculates a weighted-score based on a patient’s active comorbidities. The patient was defined as having a comorbidity condition if they had an active corresponding diagnosis code in the electronic health record problem list, and the start date of diagnosis was before 2021.

We observed differences in the proportion of patients who received any message response within 1 and 3 business days by race and ethnicity, insurance type, and primary language. In the following sections, we describe unadjusted and adjusted results for each demographic category.

### Patterns by Race and Ethnicity

**Figure 1.**
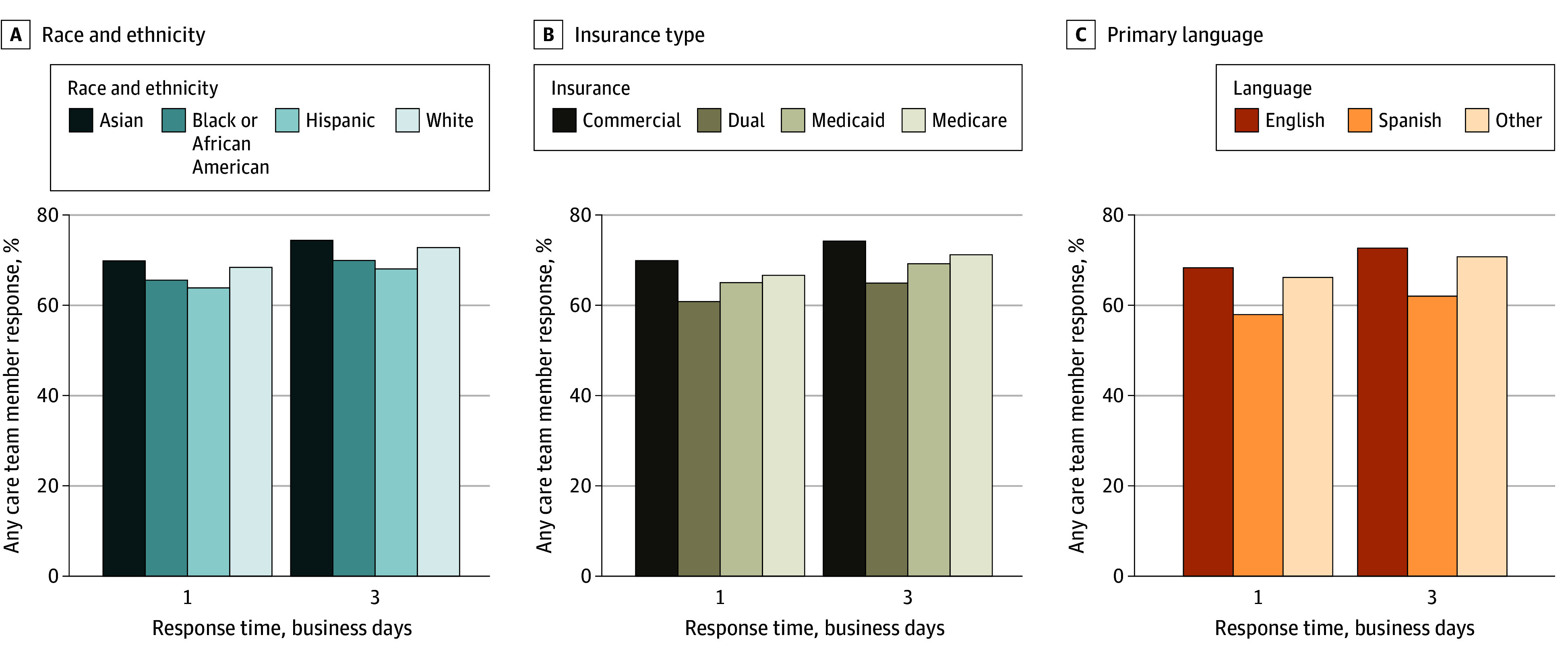
Percentage of Any Care Team Member Responses in 1 and 3 Business Days by Patient Race and Ethnicity, Insurance, and Primary Language Business days calculated using the New York Stock Exchange calendar from the 2021 year. Any response defined as any response from a care team member to the first patient message. Other race and ethnicity indicates the patient did not identify their race and ethnicity among those listed. Other patient insurance indicates that the patient had insurance listed but it was not 1 of the 4 named categories. Other language indicates that the patient’s primary language was not English or Spanish.

**Figure 2.**
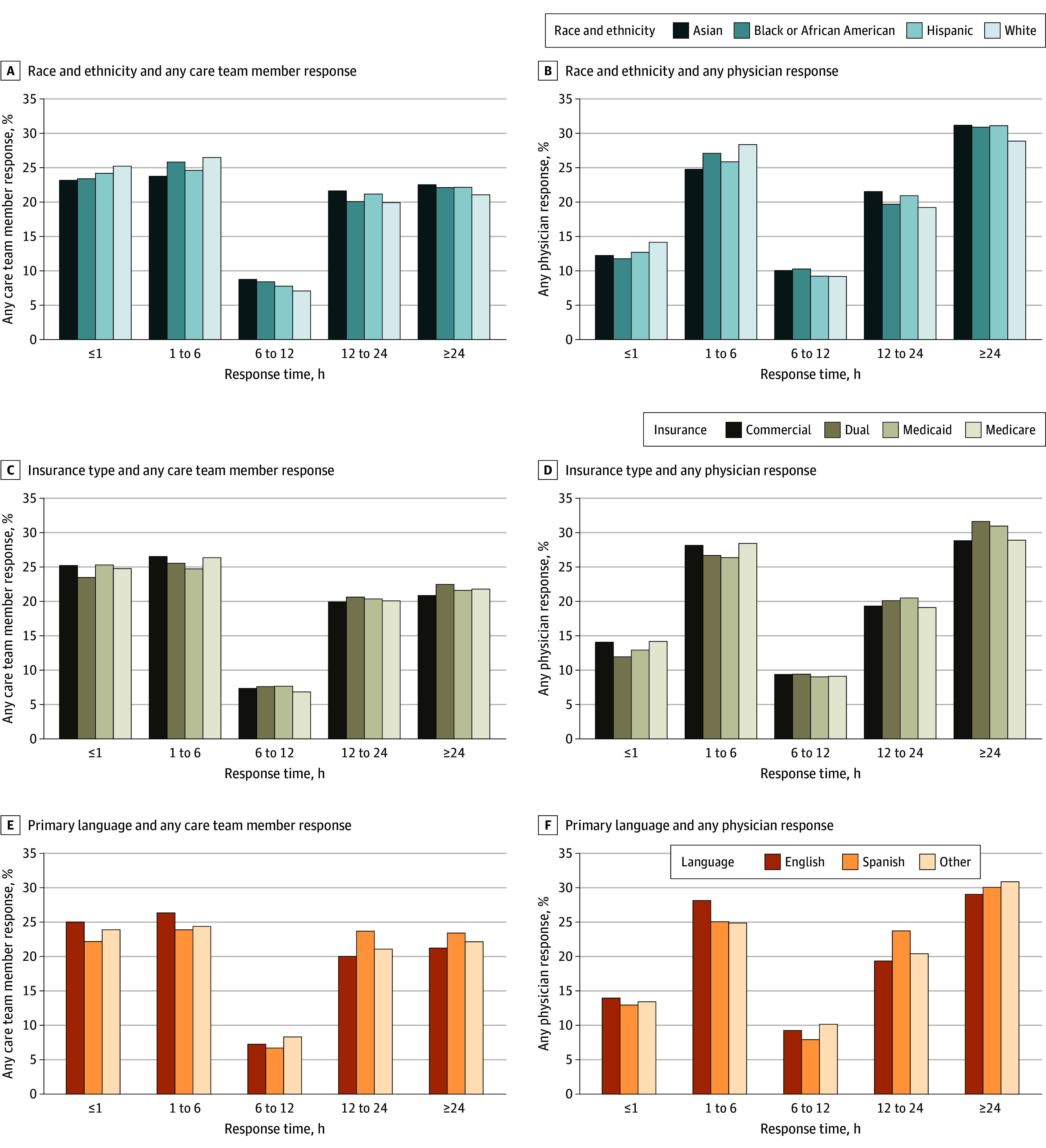
Distribution of Any Care Team Member Response Times and Physician Response Times by Patient Race and Ethnicity, Insurance, and Primary Language Other race and ethnicity indicates the patient did not identify their race and ethnicity among those listed. Other patient insurance indicates that the patient had insurance listed but it was not 1 of the 4 named categories. Other language indicates that the patient’s primary language was not English or Spanish.

While 69.9% of message threads from Asian patients (37 752 of 54 012) and 68.5% of message threads from White patients (743 161 of 1 085 517) received a response within 1 business day, 65.7% of message threads from Black or African American patients (32 165 of 48 983) and 63.9% of message threads from Hispanic or Latino patients (21 732 of 34 014) received a response within 1 business day (Figure 1A), with similar results for 3 business days. The proportion of message threads with any response from a PCP also varied by race and ethnicity (eTable 2 in Supplement 1). For example, 33.4% of message threads from White patients (362 286 of 1 085 517) received a message response from a PCP compared with 29.0% of message threads from Black or African American patients (14 220 of 48 983) and 26.0% of message threads from Hispanic patients (8842 of 34 014). The same pattern of disparities across race and ethnicity was repeated when examining time to response by any staff or PCP. Message threads from White patients (128 645 [25.3%]) had higher rates of receiving responses in less than 1 hour than message threads from Asian patients (6193 [23.2%]), Black or African American patients (5569 [23.4%]), and Hispanic or Latino patients (3976 [24.2%]) (Figure 2A and B). Across the whole distribution of response times, the median (IQR) time to first response from any care team member was 6.2 (0.8-21.1) hours for message threads from Asian patients, 5.0 (0.8-21.0) hours for Black or African American patients, 4.9 (0.8-20.5) hours for Hispanic patients, and 3.8 (0.7-20.1) hours for White patients (eTable 3A in Supplement 1). Patterns were similar for response times from PCPs (eTable 3B in Supplement 1).

Differences in the prevalence of responses within 1 business day persisted after multivariable adjustment, although many of the differences were of smaller magnitude (Table 2). Compared with White patients, in the full model with time, zip code, and clinic fixed effects, the prevalence of receiving any response within 1 business day was lower for Black patients (1.1 [95% CI, 0.2-2.0] percentage points; *P* = .01) and Hispanic patients (1.1 [95% CI, 0.3-1.9] percentage points; *P* = .01). Similar patterns were seen for the adjusted rate of a response within 3 business days (eTable 4 in Supplement 1). We also found adjusted differences in any team member (Table 3) and PCP median response times (eTable 5 in Supplement 1) to the first patient messages; however, these differences were not sustained upon full adjustment for time and clinic fixed effects. Adjusted differences were qualitatively similar when further adjusting for message content (Table 3).

**Table 2.** Adjusted Linear Probability Model for Proportion of Message Threads With Any Care Team Member Response in 1 Business Day^a^

Characteristic	Unadjusted differences in response time, min	*P* value	Adjusted differences in response time, min					
			With patient zip code and time fixed effects	*P* value	With clinic fixed effects (time and zip code fixed effects)	*P* value	For message content and clinic, time, and zip code fixed effects	*P* value
Race and ethnicity								
Asian	1.4 (1.0 to 1.8)	<.001	−0.3 (−1.4 to 0.8)	.60	0.1 (−0.5 to 0.6)	.78	0 (−0.01 to 0.01)	.98
Black or African American	−2.8 (−3.2 to −2.4)	<.001	−1.3 (−2.2 to −0.4)	<.001	−1.1 (−2.0 to −0.2)	.01	−0.01 (−0.02 to 0)	.01
Hispanic	−4.6 (−5.1 to −4.1)	<.001	−2.4 (−3.5 to −1.4)	<.001	−1.1 (−1.9 to −0.3)	.01	−0.01 (−0.02 to −0.01)	<.001
Native American or Pacific Islander	−4.6 (−6.1 to −3.2)	<.001	−2.7 (−4.6 to −0.7)	.01	−2.7 (−4.5 to −1.0)	<.001	−0.02 (−0.04 to −0.01)	.01
White	1 [Reference]		1 [Reference]		1 [Reference]		1 [Reference]	
Declined or unavailable	−1.1 (−1.7 to −0.6)	<.001	−0.9 (−1.7 to −0.2)	.02	−0.6 (−1.2 to 0.1)	.09	−0.01 (−0.01 to 0)	.02
Other^b^	−1.8 (−2.5 to −1.0)	<.001	−1.1 (−2.2 to 0.0)	.04	−1.0 (−2.0 to −0.1)	.04	−0.01 (−0.02 to 0)	.02
Insurance category								
Commercial	1 [Reference]		1 [Reference]		1 [Reference]		1 [Reference]	
Dual	−9.1 (−9.4 to −8.7)	<.001	−5.3 (−6.1 to −4.5)	<.001	−4.9 (−5.5 to −4.2)	<.001	−0.05 (−0.05 to −0.04)	<.001
Medicaid	−4.8 (−5.1 to −4.5)	<.001	−3.5 (−4.2 to −2.8)	<.001	−3.2 (−3.7 to −2.6)	<.001	−0.03 (−0.04 to −0.03)	<.001
Medicare	−3.2 (−3.4 to −3.0)	<.001	−0.9 (−1.2 to −0.5)	<.001	−0.9 (−1.2 to −0.6)	<.001	−0.01 (−0.01 to −0.01)	<.001
Other^c^	−5.0 (−6.7 to −3.2)	<.001	−2.9 (−5.7 to 0.0)	.05	−2.0 (−4.3 to 0.4)	.10	−0.01 (−0.04 to 0.01)	.26
Unavailable	−9.0 (−10.3 to −7.7)	<.001	−5.5 (−8.4 to −2.6)	<.001	−4.7 (−7.2 to −2.1)	<.001	−0.03 (−0.05 to −0.01)	.01
Language								
English	1 [Reference]		1 [Reference]		1 [Reference]		1 [Reference]	
Spanish	−10.4 (−11.2 to −9.6)	<.001	−5.7 (−7.5 to −3.9)	<.001	−4.1 (−5.7 to −2.5)	<.001	−0.04 (−0.05 to −0.02)	<.001
Other or missing^d^	−2.1 (−2.7 to −1.4)	<.001	−0.6 (−1.8 to 0.5)	.26	0.1 (−0.8 to 1.0)	.82	0.01 (0 to 0.02)	.08

^a^ Base multivariable model adjusts for patient race, sex, insurance, age, primary language, Elixhauser Score, and clinician sex, and includes patient zip code and time fixed effects, with standard errors clustered by physician. Adjusted clinic fixed effects model has the same adjustments as prior model but with clinic fixed effects included. Adjusted message content model has the same adjustments as the clinic fixed effects model but additionally adjusts for message content. Insurance defined as insurance coverage as of January 2021. Age defined as age at the beginning of 2021 based on patient’s birth date. The van Walraven weighted Elixhauser score was used which calculates a weighted-score based on a patient’s active comorbidities. The patient was defined as having a comorbidity condition if they had an active corresponding diagnosis code in the problem list, and the start date of diagnosis was prior to 2021. Day or year and hour of first message was used to apply time fixed effects.

^b^ The patient did not identify their race and ethnicity among those listed.

^c^ That the patient had insurance listed but it was not 1 of the 4 named categories.

^d^ That the patient’s primary language was not English or Spanish or was missing.

**Table 3.** Adjusted Linear Regression Model for Response Time to First Patient Message in Message Thread by Any Team Member^a^

Characteristic	Unadjusted differences in response time (95% CI), min	*P* value	Adjusted differences in response time (95% CI), min					
			With patient zip code and time fixed effects	*P* value	With clinic fixed effects (time and zip code)	*P* value	For message content and clinic, time, and zip code fixed effects	*P* value
Race and ethnicity								
Asian	96.5 (66.4 to 126.7)	<.001	30.61 (−12.58 to 73.81)	.17	30.66 (−1.62 to 62.93)	.06	27.8 (−4.4 to 59.9)	.09
Black or African American	47.2 (15.3 to 79)	<.001	5.66 (−35.71 to 47.04)	.79	2.44 (−33.97 to 38.85)	.90	−11 (−47.5 to 25.5)	.55
Hispanic	34.4 (−3.7 to 72.4)	.08	0.83 (−47.07 to 48.73)	.97	−61.04 (−102.31 to −19.76)	<.001	−72.3 (−113.6 to −30.9)	<.001
Native American and Pacific Islander	45.8 (−65.7 to 157.3)	.42	30.59 (21.32 to 154.24)	.01	23.69 (−91.6 to 138.99)	.69	21.1 (−93.8 to 136)	.72
White	1 [Reference]	NA	1 [Reference]	NA	1 [Reference]	NA	1 [Reference]	NA
Declined or unavailable	89 (47.1 to 131)	<.001	48.05 (−6.22 to 102.31)	.08	13.72 (−31.4 to 58.85)	.55	6 (−39.1 to 51)	.80
Other^b^	123.9 (66.9 to 181)	<.001	87.78 (−88.98 to 150.17)	.62	84.86 (20.18 to 149.53)	.01	75.8 (11.9 to 139.7)	.02
Insurance category								
Commercial	1 [Reference]	NA	1 [Reference]	NA	1 [Reference]	NA	1 [Reference]	NA
Dual	16.6 (−12.7 to 45.8)	.27	4.5 (−29.96 to 38.97)	.80	6.94 (- 24.54 to 38.41)	.67	−0.6 (−32 to 30.7)	.97
Medicaid	27.1 (4.2 to 50.1)	.02	18.81 (−13.33 to 50.96)	.25	17.03 (−9.11 to 43.18)	.20	12.6 (−13.4 to 38.6)	.34
Medicare	−18.7 (−33.5 to −3.9)	.01	−24.38 (−46.66 to −2.11)	.03	−26.68 (−47.7 to −5.67)	.01	−24.2 (−45.1 to −3.2)	.02
Unavailable	166.2 (57.8 to 274.6)	<.001	66.73 (−64.2 to 197.65)	.32	63.07 (−62.83 to 188.97)	.33	44.6 (−80.4 to 169.5)	.48
Other^c^	229.3 (88.5 to 370.1)	<.001	164.22 (−15.31 to 343.75)	.07	109.69 (−68.33 to 287.71)	.23	107.9 (−70.2 to 286)	.24
Language								
English	1 [Reference]	NA	1 [Reference]	NA	1 [Reference]	NA	1 [Reference]	NA
Spanish	194 (125.8 to 262.3)	<.001	177.48 (86.11 to 268.85)	<.001	78.98 (−7.14 to 165.1)	.07	85.2 (−2.1 to 172.6)	.06
Other^d^	82.1 (30.7 to 133.4)	<.001	56.87 (−9.3 to 123.04)	.09	41 (−17.62 to 99.63)	.17	27 (−31.3 to 85.3)	.36

Abbreviation: NA, not available.

^a^ Base multivariable model adjusts for patient race, sex, insurance, age, primary language, Elixhauser Score, and clinician sex, and includes patient zip code and time fixed effects, with standard errors clustered by physician. Adjusted clinic fixed effects model has the same adjustments as prior model but with clinic fixed effects included. Adjusted message content model has the same adjustments as the clinic fixed effects model but additionally adjusts for message content. Insurance defined as insurance coverage as of January 2021. Age defined as age at the beginning of 2021 based on patient’s birth date. The van Walraven weighted Elixhauser score was used which calculates a weighted-score based on a patient’s active comorbidities. The patient was defined as having a comorbidity condition if they had an active corresponding diagnosis code in the problem list, and the start date of diagnosis was prior to 2021. Day of year and hour of first message was used to apply time fixed effects.

^b^ The patient did not identify their race/ethnicity among those listed.

^c^ That the patient had insurance listed but it was not 1 of the 4 named categories.

^d^ That the patient’s primary language was not English or Spanish or was missing.

### Patterns by Insurance Coverage

Across insurance types, 70.0% of message threads from patients with commercial insurance (529 803 of 756 923) received any message reply within 1 business day compared with 65.2% of message threads from patients with Medicaid (65 154 of 99 976), 66.8% of message threads from patients with Medicare (226 517 of 339 257), and 60.9% of message threads from patients who were dual-eligible for Medicare and Medicaid (40 846 of 67 045) (Figure 1B). Meanwhile, 33.3% of message threads from patients with commercial insurance (251 713 of 756 923) received a response from a PCP, while 26.8% of message threads from patients with Medicaid (26 829 of 99 976) received a response from a PCP (eTable 2 in Supplement 1). Among those message threads from patients with commercial insurance, the median (IQR) first response time from any team member was 3.9 (0.7-19.9) hours compared with 4.5 (0.7-20.4) hours for those message threads from patients with Medicaid coverage (Figure 2C and D and eTable 3A in Supplement 1). Patterns were similar for response times from PCPs (eTable 3B in Supplement 1).

Compared with message threads from patients with commercial insurance, the rate of receiving a response within 1 business day was 4.9 (95% CI, 4.2-5.5) percentage points lower for message threads from dual-eligible patients (*P* < .001), 3.2 (95% CI, 2.6-3.7) percentage points lower for message threads from Medicaid patients (*P* < .001), and 0.9 (95% CI, 0.6-1.2) percentage points lower for message threads from Medicare patients (*P* < .001) (Table 2). In adjusted models, the differences in median response time between patients with commercial insurance and patients with other insurance became statistically insignificant after controlling for the full range of fixed effects (Table 3). Once again, adjusted differences were qualitatively similar when further adjusting for message content (Table 3).

### Patterns by Primary Language

By language spoken, 68.4% of message threads from patients who preferred English (847 489 of 1 239 768) received any reply within 1 business day, and 58.0% of message threads from patients (6898 of 11 903) who preferred Spanish received any reply within 1 business day (Figure 1C). Similar trends were seen for responses within 3 business days. For any response from a PCP, 33.0% of message threads from patients with English as their primary language (409 603 of 1 239 768) received a response from a PCP, while only 25.4% of message threads from patients with Spanish as their primary language (3018 of 11 903) received a response from a PCP (eTable 2 in Supplement 1). Among those with English as a preferred language, the median (IQR) first response time to the first message in a thread from any care team member was 4.0 (0.7-20.2) hours compared with 8.4 (1.0-22.4) hours for those message threads from patients with Spanish as a preferred language (Figure 2E and F and eTable 3A in Supplement 1). Patterns were similar for response times from PCPs (eTable 3B in Supplement 1).

In adjusted models with time, zip code, and clinic, patients whose preferred language was Spanish had a 4.1 (95% CI, 2.5-5.7) percentage point lower rate of receiving a response from any care team within 1 business day than patients whose primary language was English (*P* < .001) (Table 2). Adjusted response times for Spanish preferring patients were 36.2 (95% CI, 2.0-70.4) minutes longer than for patients with English as a preferred language, with similar findings after adjustment for clinic fixed effects (*P* = .04) (Table 3 and eTable 5 in Supplement 1). Similar results were present for the outcome of any care team response after 3 business days (eTable 4 in Supplement 1). The likelihood of any response within 1 and 3 business days (Table 2 and eTable 3 in Supplement 1) and adjusted differences in response times (Table 3) were qualitatively similar when further adjusting for message content.

### Sensitivity Analyses

In sensitivity analyses, the adjusted differences were qualitatively unchanged when accounting for primary encounters within 1 or 3 business days of a message (eTables 6 and 7 in Supplement 1). Similar results were seen for the binary outcomes of any care team response within 1 or 3 business days when specifying models for these outcomes using logistic regression rather than linear probability models (eTables 8 and 9 in Supplement 1).

## Discussion

In this cross-sectional study of primary care patients and their care teams across a large, integrated health care network, we found significant unadjusted differences in patterns of asynchronous patient-clinician communication by race and ethnicity, insurance status, and primary language. Given existing known differential care experiences by patient characteristics for care delivered in-person and for diagnostic and treatment approaches,^20,21^ our findings raise concern that electronic communication reflected disparities observed in experiences of care in other settings, particularly for patients with Medicaid insurance and who do not speak English. While many observed disparities were explained in adjusted modeling that accounted for time, clinic, and patient zip code fixed effects, our analyses demonstrated persistence of some differences, particularly for patients with Medicaid who preferred speaking Spanish. Regardless of the adjusted results, the lower response rate and slower response times demonstrate a different standard experienced by traditionally underserved groups in this health system population. This analysis highlights a new dimension of health care equity that health systems should consider measuring and understanding to improve disparities in patient experiences.

While the exact causes of the differences we identified cannot be precisely discerned, our models suggest a likely role of differential structures and workflows present in the clinics caring for patients from minoritized racial and ethnic groups, patients who have Medicaid, and patients who do not prefer English. Specifically, there was a notable attenuation of differences in the percentage of patients receiving a response at 1 and 3 business days and response times with the inclusion of time, clinic, and zip code fixed effects. This attenuation has 2 main implications. First, it suggests that clinics more likely to care for patients from minoritized racial and ethnic groups, patients who do not have commercial insurance, and patients who do not prefer English were less likely to have the resourcing or workflows to ensure fast responses to asynchronous patient messages or to meet patients’ messaging-related language access needs in a timely fashion. This could be due to staffing constraints due to funding differences or workflows that were focused on alternative priorities, such as in-person services, but other explanations are possible. Second, it suggests that different patient groups have different patterns of when they send messages. For example, patients with Medicaid may have less flexibility with access to messaging during the business day, pushing more communication to nights and weekends.

While our evidence is from a single health system, evidence from national data sources suggest that asynchronous patient messages have generated new task loads for primary care clinics that are already overburdened.^22,23^ Although much attention to patient messaging has emphasized workforce well-being, our results emphasize its importance for the delivery of equitable care as well. This will be important to monitor as generative artificial intelligence becomes more integrated into patient messaging.^24^ On the one hand, artificial intelligence-based solutions could improve response time disparities, perhaps by drafting messages in languages other than English, but it could also lead to unanticipated biases (eg, differential responses based on patient characteristics, suboptimal interpretation into non-English languages). The specific circumstances of the health system in this study may not generalize elsewhere, but disparities in patient messaging are likely common across health systems.

### Limitations and Strengths

Our study has several limitations. First, it reflects patterns of asynchronous primary care delivery across a large, integrated health care system in the northeastern United States that includes 2 academic medical centers. While this represents nearly 800 000 patients and their primary care teams, it may not reflect the experiences of patients receiving care in other parts of the country or through other health care models. The present analyses only focused on messaging between patients and their primary care teams. Therefore, it may not reflect the experiences of patients receiving asynchronous care from specialists. Additionally, while we identified differences in the likelihood of any response, response within 1 or 3 business days, or time to first response by patient characteristics, there is not a clearly established association of these metrics with patient outcomes. Another limitation is that the data are from 2021, which was during the COVID-19 pandemic and reflects a time of differing health care operations. Therefore, these results may not directly generalize to health systems in 2025 and beyond. Finally, the detection of significant differences in response rates and times may be driven in part by stochastic variation in our large sample size of PCPs and patients and not systemic bias. Balancing these limitations are several strengths. First, our use of detailed EHR data provides insight into message response patterns in the clinical setting and a more granular level of understanding regarding asynchronous messaging patterns and responses than possible with previous studies. The large sample size of patients and PCPs represented in this study enables us to draw conclusions across patient demographic groups and clinics. Finally, we differentiate responses to patient messages by care team member and PCPs, deepening our understanding of how different care team members interface with asynchronous messaging.

## Conclusions

In conclusion, in this study of messaging patterns across a large, integrated health care network, we demonstrated slower message response times among patients from minoritized racial and ethnic groups, patients who do not have commercial insurance, and patients who do not prefer speaking English. These differences were partially explained by time and clinic-specific differences after adjustment, suggesting resource gaps among clinics who served these populations. As the prevalence of EHR-based, asynchronous delivery expands across the US, our findings underscore the importance of adequately equipping and resourcing clinics and care teams who care for underserved populations via EHR-based care modalities to avoid perpetuation of known health care disparities.

## Notes

Supplement 1. **eMethods. **Message Content Classification **eFigure. ** Cohort Flow Diagram **eTable 1.** Characteristics of Patients in Sample by Messages and Threads **eTable 2.** Distribution of Messages with Any Response from a PCP for by Patient Race/Ethnicity, Insurance, and Language **eTable 3. **First Response Time by Any Care Team Member or Primary Care Physician **eTable 4. **Adjusted Linear Probability Models for Proportion of Messages with any Response in 3 Business Day **eTable 5. **Adjusted Linear Regression Models for Response Time to First Patient Message by Primary Care Physician **eTable 6. **Adjusted Linear Probability Models for Any Response or Encounter in 1 Business Day **eTable 7. **Adjusted Linear Probability Model for Any Response or Encounter in 3 Business Days **eTable 8.** Adjusted Logistic Regression Models for Proportion of Messages with Any Care Team Member Response in 1 Business Day by Patient Characteristics **eTable 9. **Adjusted Logistic Regression Models for Proportion of Messages with Any Care Team Member Response in 3 Business Days by Patient Characteristics

Supplement 2.
Data Sharing Statement
