# Supplementary material for: Patient-Physician Messaging by Race, Ethnicity, Insurance Type, and Preferred Language
Source: JAMA Netw Open. 2025 Oct 7;8(10):e2534549. doi: 10.1001/jamanetworkopen.2025.34549 (PMC12505178; doi:10.1001/jamanetworkopen.2025.34549)
Supplement: Supplement 1. — eMethods. Message Content Classification eFigure. Cohort Flow Diagram eTable 1. Characteristics of Patients in Sample by Messages and Threads eTable 2. Distribution of Messages with Any Response from a PCP for by Patient Race/Ethnicity, Insurance, and Language eTable 3. First Response Time by Any Care Team Member or Primary Care Physician eTable 4. Adjusted Linear Probability Models for Proportion of Messages with any Response in 3 Business Day eTable 5. Adjusted Linear Regression Models for Response Time to First Patient Message by Primary Care Physician eTable 6. Adjusted Linear Probability Models for Any Response or Encounter in 1 Business Day eTable 7. Adjusted Linear Probability Model for Any Response or Encounter in 3 Business Days eTable 8. Adjusted Logistic Regression Models for Proportion of Messages with Any Care Team Member Response in 1 Business Day by Patient Characteristics eTable 9. Adjusted Logistic Regression Models for Proportion of Messages with Any Care Team Member Response in 3 Business Days by Patient Characteristics [file jamanetwopen-e2534549-s001.pdf]

Supplemental Online Content

Rotenstein LS, Hardy B, Tang M, et al. Patient-physician messaging by race, ethnicity, and insurance type. *JAMA Netw Open*. 2025;8(9):e2534549. doi:10.1001/jamanetworkopen.2025.34549

eMethods. Message Content Classification

eFigure. Cohort Flow Diagram

eTable 1. Characteristics of Patients in Sample by Messages and Threads

eTable 2. Distribution of Messages with Any Response from a PCP for by Patient Race/Ethnicity, Insurance, and Language

eTable 3. First Response Time by Any Care Team Member or Primary Care Physician

eTable 4. Adjusted Linear Probability Models for Proportion of Messages With Any Response in 3 Business Day

eTable 5. Adjusted Linear Regression Models for Response Time to First Patient Message by Primary Care Physician

eTable 6. Adjusted Linear Probability Models for Any Response or Encounter in 1 Business Day

eTable 7. Adjusted Linear Probability Model for Any Response or Encounter in 3 Business Days

eTable 8. Adjusted Logistic Regression Models for Proportion of Messages with Any Care Team Member Response in 1 Business Day by Patient Characteristics

eTable 9. Adjusted Logistic Regression Models for Proportion of Messages with Any Care Team Member Response in 3 Business Days by Patient Characteristics

This supplemental material has been provided by the authors to give readers additional information about their work.

© 2025 Rotenstein LS et al. *JAMA Network Open*.

eMethods: Message Content Classification

Message threads in our sample were categorized into one of twenty topic groups using a latent Dirichlet allocation (LDA) model based on the text of the initial patient message in the thread. Implementation of the LDA model was done using the python gensim package (link: https://pypi.org/project/gensim/).

Within the LDA model, the text of each initial patient message was represented as a "bag of words", reflecting the frequency of individual words and word bigrams (combinations of two words, e.g., nurse practitioner) comprising the message. Notably, these representations ignore word ordering and broader context (outside of word pairs captured in bigrams). Common "stop words" (e.g., "is", "in", "for", "where") were identified using the Natural Language Toolkit (nltk) python package (link: https://www.nltk.org/) and excluded from these representations. Additionally, all words were converted to root forms (lemmas) utilizing the spacy python package (link: https://pypi.org/project/spacy/), such as converting plural nouns to singular, and verb conjugations to their base verbs.

The LDA model takes as an input a target number of topic groups and a training set of text documents (messages in this case) pre-processed as described previously. Through the training process, the model identifies, in an unsupervised manner, a specific set of topic groups across the training set along with specific keywords and phrases associated with each topic.

Our LDA model was trained on a random subset of 1 million patient medical advice request message threads sent to primary care departments in the Mass General Brigham health system from 2021. The twenty topics resulting from our LDA model are shown below. Topic category labels were defined ex-post based on review of topic keywords and example messages assigned to that topic label.

Topic category	Key words
Referral	appointment, schedule, thank, referral, need, see, visit, make, follow, physical
Form request	need, letter, thank, form, send, medical, record, work, fax, note
Insurance-related	insurance, cover, pay, company, bill, plan, say, health, cost, require
Booking related update	know, let, get, call, go, want, thank, today, say, see
New patient	care, patient, new, doctor, year, take, hospital, primary, urgent, gateway
COVID-19	test, covid, positive, negative, symptom, home, result, get, thank, day
Blood work	blood, test, result, thank, lab, work, order, do, pressure, see
Concerning lab result	result, issue, follow, see, concern, report, say, also, note, regard
Medication refill	prescription, refill, thank, send, need, pharmacy, day, new, request, get
Medication symptom update	take, day, night, sleep, stop, medication, start, morning, thank, last
Antibiotics	antibiotic, infection, urine, prescribe, symptom, thank, finish, treat, oral, burn
Proxy message	send, message, behalf, receive, thank, sleep, study, reply, brand, name
Seeking help	help, well, thank, hope, get, good, much, try, see, work

© 2025 Rotenstein LS et al. *JAMA Network Open*.

Longitudinal update	time, get, week, feel, go, last, day, month, think, ago
Cold and flu-related symptoms	cough, feel, throat, fever, get, day, symptom, still, well, ear
Pain - group 1	pain, back, get, knee, see, walk, help, go, thank, hip
Pain - group 2	right, pain, leave, side, feel, hand, foot, arm, leg, area
Skin or eye infection or rash	eye, see, attach, skin, thank, picture, rash, look, get, use
Weight	weight, increase, lose, high, loss, low, dose, diet, level, eat
Vaccination	vaccine, get, covid, booster, shot, receive, thank, shoot, second, vaccination

Our trained LDA model was then applied to the 2021 message threads included in our study sample. The text of each thread's initiating message went through our pre-processing steps described earlier. Using the LDA model, each thread was assigned a vector of topic probabilities across our 20 potential topic groups. Each message thread was then assigned to the single "dominant" topic group that had the highest probability score. These topic group labels were then included as fixed effect controls in additional sensitivity regression analyses to determine if message content might account for some of the observed differences in response outcomes across patient demographic groups.

© 2025 Rotenstein LS et al. *JAMA Network Open*.

eFigure 1: Cohort Flow Diagram

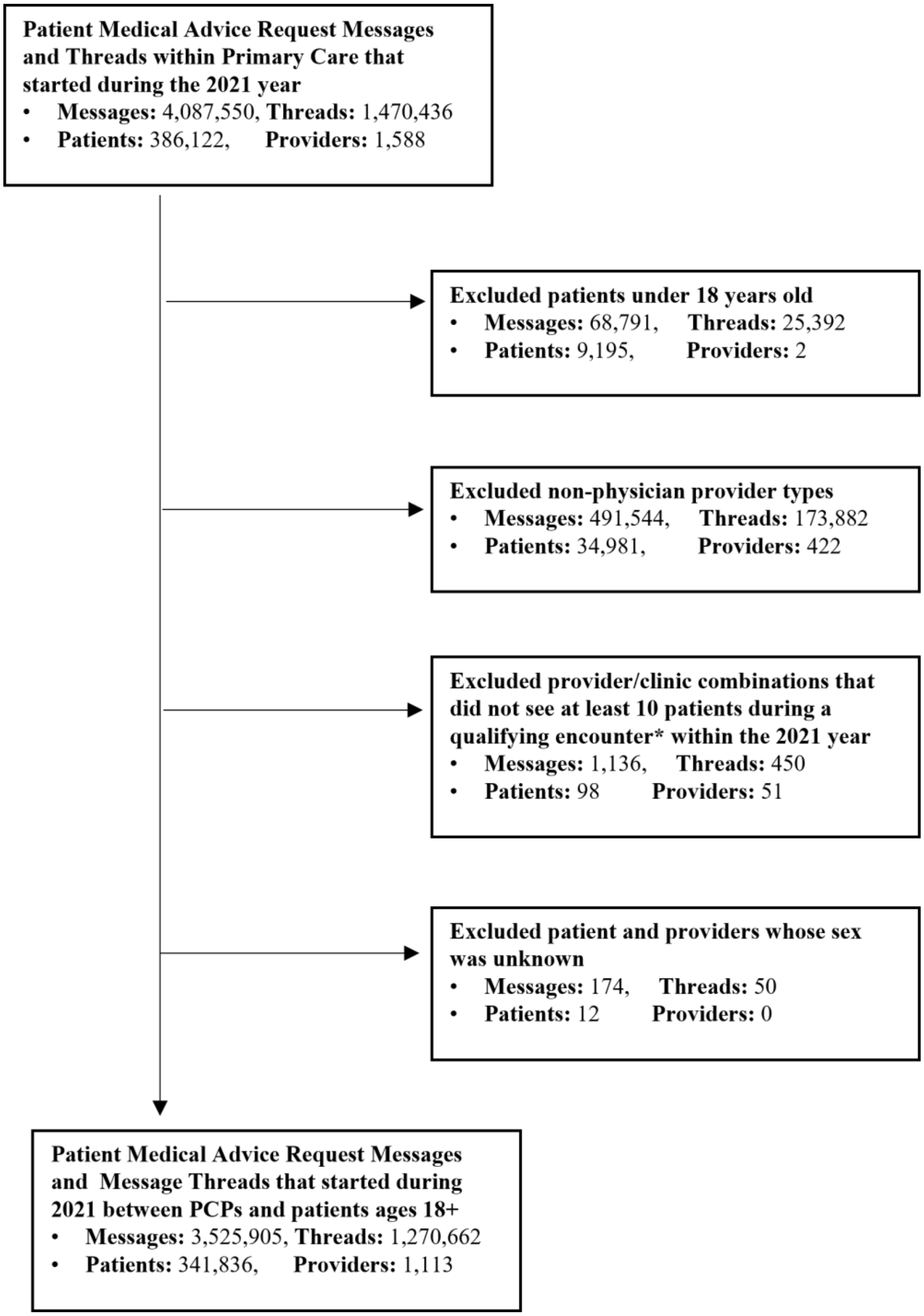

Note: Age was calculated as age at the beginning of the 2021 year using date of birth. A qualifying encounter was defined as any office or telehealth visit. Patient or provider sex listed as “X”, “Unknown” or missing was excluded.

© 2025 Rotenstein LS et al. *JAMA Network Open*.

eTable 1: Characteristics of Patients in Sample by Messages and Threads

	All Messages		All Threads	
	N	%	N	%
**Patient Race**				
White	3,003,493	85.2%	1,085,517	85.4%
Asian	155,589	4.4%	54,012	4.3%
Black or African American	137,075	3.9%	48,983	3.9%
Declined or Unavailable	80,815	2.3%	28,979	2.3%
Hispanic	94,254	2.7%	34,014	2.7%
Native American or Pacific Islander	11,198	0.3%	4021	0.3%
Other*	43,481	1.2%	15,136	1.2%
**Patient Insurance**				
Commercial	2,154,290	61.1%	756,923	59.6%
Medicaid	284,279	8.1%	99,976	7.9%
Medicare	889,914	25.2%	339,257	26.7%
Dual	177,158	5.0%	67,045	5.3%
Other**	7,382	0.2%	2698	0.2%
Unavailable	12,882	0.4%	4,763	0.4%
**Age**				
18-27	276,079	7.8%	93,827	7.4%
28-37	450,752	12.8%	151,709	11.9%
38-47	463,636	13.1%	159,749	12.6%
48-57	644,697	18.3%	229,966	18.1%
58-67	793,692	22.5%	291,455	22.9%
68-79	704,955	20.0%	269,092	21.2%
80+	192,094	5.4%	74,864	5.9%
**Sex**				
Female	2,258,503	64.1%	808,957	63.7%
Male	1,267,402	35.9%	461,705	36.3%
**Primary Language**				
English	3,443,881	97.7%	1,239,768	97.6%
Spanish	29,224	0.8%	11,903	0.9%
Other/Missing***	52,800	1.5%	18,991	1.5%
**Elixhauser Score**				
Mean (Std Dev)	2.7 (7.01)		2.87 (7.16)	

*Indicates the patient did not identify their race/ethnicity among those listed. **Indicates that the patient had insurance listed but it was not one of the four named categories. ***Indicates that the patient's primary language was not English/Spanish or was missing.

Note: The Van Walraven weighted Elixhauser score was used which calculates a weighted- score based on a patient's active comorbidities. The patient was defined as having a comorbidity condition if they had an active corresponding diagnosis code in the Problem List, and the start date of diagnosis was prior to the 2021 year. Also note that the "Native American or Pacific Islander Category" also includes "American Indian or Alaska Native" coded individuals.

© 2025 Rotenstein LS et al. *JAMA Network Open*.

eTable 2: Distribution of Messages with Any Response from a PCP for by Patient Race/Ethnicity, Insurance, and Language

	Threads with PCP Response	Total Threads	% PCP Response
**Race/Ethnicity**			
Asian	17,837	54,012	33.0%
Black or African American	14,220	48,983	29.0%
Hispanic	8,842	34,014	26.0%
White	362,286	1,085,517	33.4%
Other*	4,751	15,136	31.4%
**Insurance Category**			
Commercial	251,713	756,923	33.3%
Dual	18,971	67,045	28.3%
Medicaid	26,829	99,976	26.8%
Medicare	118,700	339,257	35.0%
Other**	922	2,698	34.2%
**Language**			
English	409,603	1,239,768	33.0%
Spanish	3,018	11,903	25.4%
Other***	5,996	18,991	31.6%

*Indicates the patient did not identify their race/ethnicity among those listed. **Indicates that the patient had insurance listed but it was not one of the four named categories. ***Indicates that the patient's primary language was not English/Spanish or was missing.

© 2025 Rotenstein LS et al. *JAMA Network Open*.

eTable 3: First Response Time by Any Care Team Member or Primary Care Physician

A. First Response Time by Any Care Team Member (Hours)

	Median	IQR
**Race**		
Asian	6.2	(0.8, 21.1)
Black or African American	5.0	(0.8, 21.0)
Hispanic	4.9	(0.8, 20.5)
White	3.8	(0.7, 20.1)
Other*	5.4	(0.8, 21.1)
**Insurance Type**		
Commercial	3.9	(0.7, 19.9)
Medicare	3.9	(0.7, 20.6)
Medicaid	4.5	(0.7, 20.4)
Dual	4.6	(0.8, 20.4)
Other**	4.0	(0.6, 22.1)
**Primary Language**		
English	4.0	(0.7, 20.2)
Spanish	8.4	(1.0, 22.4)
Other***	5.0	(0.8, 20.6)

*Indicates the patient did not identify their race/ethnicity among those listed. **Indicates that the patient had insurance listed but it was not one of the four named categories. ***Indicates that the patient's primary language was not English/Spanish or was missing.

B. First Response Time by a Primary Care Physician (Hours)

	Median	IQR
**Race**		
Asian	13.6	(2.6, 31.8)
Black or African American	12.4	(2.6, 30.2)
Hispanic	13.5	(2.6, 31.0)
White	10.5	(2.1, 27.7)
Other*	12.5	(2.4, 31.4)
**Insurance Type**		
Commercial	10.5	(2.2, 27.7)
Medicare	10.5	(2.1, 27.7)
Medicaid	13.1	(2.5, 31.4)
Dual	13.1	(2.6, 32.4)
Other**	13.5	(2.9, 38.6)
**Primary Language**		
English	10.8	(2.2, 28.0)
Spanish	14.5	(2.7, 30.3)
Other***	13.3	(2.9, 38.6)

*Indicates the patient did not identify their race/ethnicity among those listed. **Indicates that the patient had insurance listed but it was not one of the four named categories. ***Indicates that the patient's primary language was not English/Spanish or was missing.

eTable 4: Adjusted Linear Probability Models for Proportion of Messages with any Response in 3 Business Day

	Unadjusted		Adjusted with Patient Zip Code and Time Fixed Effects		Adjusted with Clinic Fixed Effects (+Time and Zip Code Fixed Effects)		Adjusted for Message Content + Clinic, Time, and Zip Code Fixed Effects	
	Differences in Percent of Messages (95% CI)	p-value	Differences in Percent of Messages (95% CI)	p-value	Differences in Percent of Messages (95% CI)	p-value	Differences in Percent of Messages (95% CI)	p-value
**Race/Ethnicity**								
White	Ref		Ref		Ref		Ref	
Asian	1.6 (1.2, 1.9)	<0.001	-0.1 (-0.9, 0.7)	0.85	0.2 (-0.4, 0.7)	0.57	0 (0, 0.01)	0.76
Black/African American	-2.8 (-3.2, -2.4)	<0.001	-1.3 (-2.1, -0.4)	<0.001	-1.1 (-1.9, -0.3)	0.01	-0.01 (-0.02, 0)	0.01
Declined or Unavailable	-1 (-1.5, -0.5)	<0.001	-0.9 (-1.6, -0.1)	0.02	-0.6 (-1.2, 0.1)	0.09	-0.01 (-0.02, 0)	0.01
Hispanic	-4.7 (-5.2, -4.2)	<0.001	-2.4 (-3.3, -1.4)	<0.001	-1.3 (-2.1, -0.5)	<0.001	-0.02 (-0.02, -0.01)	<0.001
Native American/Pacific Islander	-4.4 (-5.7, -3)	<0.001	-2.4 (-4.3, -0.5)	0.01	-2.3 (-4.1, -0.6)	0.01	-0.02 (-0.04, 0)	0.02
Other*	-1.9 (-2.6, -1.2)	<0.001	-1.2 (-2.3, -0.2)	0.02	-1.1 (-2.1, -0.2)	0.02	-0.01 (-0.02, 0)	0.01
**Insurance Category**								
Commercial	Ref		Ref		Ref		Ref	
Dual	-9.3 (-9.7, -9)	<0.001	-5.4 (-6.2, -4.7)	<0.001	-5.1 (-5.8, -4.5)	<0.001	-0.05 (-0.05, -0.04)	<0.001
Medicaid	-5 (-5.3, -4.7)	<0.001	-3.7 (-4.3, -3)	<0.001	-3.3 (-3.9, -2.8)	<0.001	-0.03 (-0.04, -0.03)	<0.001
Medicare	-3.1 (-3.3, -3)	<0.001	-0.9 (-1.3, -0.6)	<0.001	-1 (-1.3, -0.6)	<0.001	-0.01 (-0.01, -0.01)	<0.001
Other**	-4.8 (-6.5, -3.2)	<0.001	-3.5 (-6.5, -0.5)	0.02	-3 (-5.6, -0.3)	0.03	-0.02 (-0.05, 0)	0.08
Unavailable	-8.5 (-9.7, -7.2)	<0.001	-5.4 (-8.2, -2.5)	<0.001	-4.8 (-7.3, -2.2)	<0.001	-0.03 (-0.05, -0.01)	0.01
**Language**								
English	Ref		Ref		Ref		Ref	
Spanish	-10.6 (-11.4, -9.8)	<0.001	-5.5 (-7.3, -3.8)	<0.001	-4.3 (-5.9, -2.6)	<0.001	-0.04 (-0.06, -0.02)	<0.001
Other/Missing***	-1.9 (-2.5, -1.2)	<0.001	-0.3 (-1.4, 0.7)	0.51	0.3 (-0.5, 1.2)	0.47	0.01 (0, 0.02)	0.04

*Indicates the patient did not identify their race/ethnicity among those listed. **Indicates that the patient had insurance listed but it was not one of the four named categories. ***Indicates that the patient's primary language was not English/Spanish or was missing.

Note: Base multivariable model adjusts for patient race, sex, insurance, age, primary language, Elixhauser Score, and physician sex, and includes patient Zip code and time fixed effects, with standard errors clustered by physician. Adjusted clinic fixed effects model has the same adjustments as prior model but with clinic fixed effects included. Adjusted message content model has the same adjustments as the clinic fixed effects model but additionally adjusts for message content. Insurance defined as insurance coverage as of January 2021. Age defined as age at the beginning of 2021 based on patient's birth date. The van Walraven weighted Elixhauser score was used which calculates a weighted- score based on a patient's active comorbidities. The patient was defined as having a comorbidity condition if they had an active corresponding diagnosis code in the problem list, and the start date of diagnosis was prior to the 2021 year.

© 2025 Rotenstein LS et al. *JAMA Network Open*.

eTable 5: Adjusted Linear Regression Models for Response Time to First Patient Message by Primary Care Physician

	Unadjusted		Adjusted with Patient Zip Code and Time Fixed Effects		Adjusted with Clinic Fixed Effects (+Time and Zip Code Fixed Effects)		Adjusted for Message Content + Clinic, Time, and Zip Code Fixed Effects	
	Differences in Message Response Time (95% CI)	p-value	Differences in Message Response Time (95% CI)	p-value	Differences in Message Response Time (95% CI)	p-value	Differences in Message Response Time (95% CI)	p-value
**Race/Ethnicity**								
White	Ref		Ref		Ref		Ref	
Asian	130.9 (86.7, 175)	<.001	111.25 (-4.95, 227.45)	0.06	63.09 (-26.29, 152.46)	0.17	31.5 (-23.2, 86.1)	0.26
Black/African American	123.7 (74.5, 172.9)	<.001	82.81 (-31.21, 196.83)	0.15	107.59 (-0.6, 215.78)	0.05	57.6 (-23.5, 138.6)	0.16
Declined or Unavailable	50.2 (-9.7, 110.1)	0.10	89.06 (-42.93, 221.04)	0.19	102.35 (-22.79, 227.49)	0.11	21.7 (-42, 85.3)	0.50
Hispanic	150.6 (88.7, 212.6)	<.001	40.62 (-118.12, 199.37)	0.62	21.72 (-98.28, 141.72)	0.72	11.1 (-72.1, 94.3)	0.79
Native American or Pacific Islander	174.8 (7.7, 341.8)	0.04	61.8 (-191.46, 315.06)	0.63	76.04 (-158.64, 310.72)	0.53	97.4 (-74.8, 269.6)	0.27
Other*	182 (97.9, 266.1)	<.001	365.84 (74.33, 657.36)	0.01	354.94 (68.49, 641.38)	0.02	94.9 (-5.4, 195.1)	0.06
**Insurance Category**								
Commercial	-						-	
Dual	119.4 (76.1, 162.8)	<.001	72.35 (-23.92, 168.61)	0.14	-13.75 (-95.21, 67.71)	0.74	18.2 (-37.3, 73.8)	0.52
Medicaid	128.9 (91.9, 165.9)	<.001	45.1 (-38.43, 128.63)	0.29	6.17 (-64.02, 76.37)	0.86	8.7 (-36.7, 54)	0.71
Medicare	-51.8 (-72.1, -31.5)	<.001	-38.14 (-88.12, 11.83)	0.14	-59.27 (-105.95, -12.58)	0.01	-31.5 (-60.9, -2.2)	0.04
Unavailable	117.1 (-32.9, 267.1)	0.13	104.08 (-206.6, 414.75)	0.51	37.86 (-246.69, 322.42)	0.79	8.9 (-158.9, 176.6)	0.92
Other**	378.7 (188.7, 568.6)	<.001	215.69 (-123.46, 554.84)	0.21	190.61 (-116.76, 497.98)	0.22	179.3 (-36.4, 395)	0.10
**Language**								
English	-						-	
Spanish	166.3 (61.1, 271.5)	<.001	44.87 (-214.2, 303.94)	0.73	31.76 (-180.82, 244.34)	0.77	81.1 (-66.4, 228.6)	0.28
Other***	94.8 (19.9, 169.7)	0.01	59.88 (-119.85, 239.61)	0.51	28.24 (-134.43, 190.92)	0.73	22.6 (-68, 113.3)	0.62

*Indicates the patient did not identify their race/ethnicity among those listed. **Indicates that the patient had insurance listed but it was not one of the four named categories. ***Indicates that the patient's primary language was not English/Spanish or was missing. Note: Base multivariable model adjusts for patient race, sex, insurance, age, primary language, Elixhauser Score, and physician sex, and includes patient Zip code and time fixed effects, with standard errors clustered by physician. Adjusted clinic fixed effects model has the same adjustments as prior model but with clinic fixed effects included. Adjusted message content model has the same adjustments as the clinic fixed effects model but additionally adjusts for message content. Insurance defined as insurance coverage as of January 2021. Age defined as age at the beginning of 2021 based on patient's birth date. The van Walraven weighted Elixhauser score was used which calculates a weighted- score based on a patient's active comorbidities. The patient was defined as having a comorbidity condition if they had an active corresponding diagnosis code in the problem list, and the start date of diagnosis was prior to the 2021 year.

Note: Response time values were top-coded at the 99th percentile at 18,880 minutes.

© 2025 Rotenstein LS et al. *JAMA Network Open*.

eTable 6: Adjusted Linear Probability Models for Any Response or Encounter in 1 Business Day

	Unadjusted		Adjusted with Patient Zip Code and Time Fixed Effects		Adjusted with Clinic Fixed Effects (+Time and Zip Code Fixed Effects)		Adjusted for Message Content + Clinic, Time, and Zip Code Fixed Effects	
	Differences in Percent of Messages (95% CI)	p-value	Differences in Percent of Messages (95% CI)	p-value	Differences in Percent of Messages (95% CI)	p-value	Differences in Percent of Messages (95% CI)	p-value
**Race/Ethnicity**								
White	Ref		Ref		Ref		Ref	
Asian	1.2 (0.8, 1.5)	<.001	0.1 (-0.4, 0.6)	0.70	0.1 (-0.4, 0.6)	0.7	0 (0, 0.01)	0.7
Black/African American	-2.6 (-3, -2.2)	<.001	-1 (-1.8, -0.1)	0.02	-1 (-1.8, -0.1)	0.02	-0.01 (-0.02, 0)	0.03
Declined or Unavailable	-1.5 (-2, -0.9)	<.001	-0.4 (-1.1, 0.2)	0.18	-0.4 (-1.1, 0.2)	0.18	0 (-0.01, 0)	0.26
Hispanic	-4.3 (-4.8, -3.8)	<.001	-1 (-1.8, -0.2)	0.01	-1 (-1.8, -0.2)	0.01	-0.01 (-0.02, 0)	<.001
Native American/Pacific Islander	-3.5 (-4.9, -2.1)	<.001	-2 (-3.8, -0.1)	0.04	-0.8 (-1.7, 0.1)	0.09	-0.02 (-0.04, 0)	0.09
Other*	-1.7 (-2.4, -0.9)	<.001	-0.8 (-1.7, 0.1)	0.09	-2 (-3.8, -0.1)	0.04	-0.01 (-0.02, 0)	0.1
**Insurance Category**								
Commercial	Ref		Ref		Ref		Ref	
Dual	-7.7 (-8.1, -7.4)	<.001	-4.2 (-4.9, -3.6)	<.001	-4.2 (-4.9, -3.6)	<.001	-0.04 (-0.05, -0.03)	<.001
Medicaid	-4 (-4.3, -3.7)	<.001	-2.8 (-3.3, -2.2)	<.001	-2.8 (-3.3, -2.2)	<.001	-0.03 (-0.03, -0.02)	<.001
Medicare	-3.2 (-3.4, -3)	<.001	-0.8 (-1.1, -0.5)	<.001	-0.8 (-1.1, -0.5)	<.001	-0.01 (-0.01, 0)	<.001
Other**	-5.6 (-7.4, -3.9)	<.001	-2.1 (-4.4, 0.2)	0.08	-2.1 (-4.4, 0.2)	0.08	-0.02 (-0.04, 0)	0.12
Unavailable	-8.6 (-9.9, -7.3)	<.001	-4.2 (-6.7, -1.6)	<.001	-2.1 (-4.4, 0.2)	0.08	-0.04 (-0.06, -0.01)	0.01
**Language**								
English	Ref		Ref		Ref		Ref	
Spanish	-9.6 (-10.5, -8.8)	<.001	-3.7 (-5.2, -2.2)	<.001	-3.7 (-5.2, -2.2)	<.001	-0.04 (-0.05, -0.02)	<.001
Other/Missing***	-2.1 (-2.7, -1.4)	<.001	0.1 (-0.8, 1)	0.80	0.1 (-0.8, 1)	0.8	0 (0, 0.01)	0.29

*Indicates the patient did not identify their race/ethnicity among those listed. **Indicates that the patient had insurance listed but it was not one of the four named categories. ***Indicates that the patient's primary language was not English/Spanish or was missing.

Note: Base multivariable model adjusts for patient race, sex, insurance, age, primary language, Elixhauser Score, and physician sex, and includes patient clinic, zip code and time fixed effects, with standard errors clustered by physician. Adjusted message content model has the same adjustments as the base model but additionally adjusts for message content Insurance defined as insurance coverage as of January 2021. Age defined as age at the beginning of 2021 based on patient's birth date. The van Walraven weighted Elixhauser score was used which calculates a weighted- score based on a patient's active comorbidities. The patient was defined as having a comorbidity condition if they had an active corresponding diagnosis code in the problem list, and the start date of diagnosis was prior to the 2021 year.

© 2025 Rotenstein LS et al. *JAMA Network Open*.

eTable 7: **Adjusted Linear Probability Model for Any Response or Encounter in 3 Business Days**

	**Unadjusted**		**Adjusted with Clinic Fixed Effects (+Time and Zip Code Fixed Effects)**		**Adjusted for Message Content + Clinic, Time, and Zip Code Fixed Effects**	
	Differences in Percent of Messages (95% CI)	p-value	Differences in Percent of Messages (95% CI)	p-value	Differences in Percent of Messages (95% CI)	p-value
**Race/Ethnicity**						
White	Ref		Ref		Ref	
Asian	1.2 (0.8, 1.6)	<.001	0.2 (-0.3, 0.7)	0.41	0 (0, 0.01)	0.46
Black/African American	-2.5 (-2.9, -2.1)	<.001	-1 (-1.8, -0.2)	0.02	-0.01 (-0.02, 0)	0.01
Declined or Unavailable	-1.4 (-1.9, -0.9)	<.001	-0.5 (-1.1, 0.2)	0.15	0 (-0.01, 0)	0.19
Hispanic	-4.2 (-4.7, -3.8)	<.001	-1.1 (-1.9, -0.3)	0.01	-0.01 (-0.02, -0.01)	<.001
Native American/Pacific Islander	-2.7 (-4.1, -1.4)	<.001	-1.3 (-3.1, 0.6)	0.18	-0.01 (-0.03, 0.01)	0.35
Other*	-1.8 (-2.5, -1.1)	<.001	-0.9 (-1.8, 0)	0.05	-0.01 (-0.02, 0)	0.05
**Insurance Category**						
Commercial	Ref		Ref		Ref	
Dual	-7.4 (-7.78, -7.1)	<.001	-4.2 (-4.9, -3.6)	<.001	-0.04 (-0.05, -0.03)	<.001
Medicaid	-3.8 (-4.12, -3.6)	<.001	-2.8 (-3.3, -2.3)	<.001	-0.03 (-0.03, -0.02)	<.001
Medicare	-3 (-3.17, -2.8)	<.001	-0.8 (-1.1, -0.5)	<.001	-0.01 (-0.01, 0)	<.001
Other**	-5.6 (-7.22, -4)	<.001	-3 (-5.7, -0.4)	0.02	-0.03 (-0.05, 0)	0.04
Unavailable	-7.9 (-9.09, -6.6)	<.001	-3.9 (-6.4, -1.4)	0.00	-0.03 (-0.06, -0.01)	0.01
**Language**						
English	Ref		Ref		Ref	
Spanish	-9.6 (-10.4, -8.9)	<.001	-4 (-5.4, -2.5)	<.001	-0.04 (-0.05, -0.02)	<.001
Other/Missing***	-1.9 (-2.5, -1.3)	<.001	0.3 (-0.5, 1.1)	0.46	0.01 (0, 0.01)	0.12

*Indicates the patient did not identify their race/ethnicity among those listed. **Indicates that the patient had insurance listed but it was not one of the four named categories. ***Indicates that the patient's primary language was not English/Spanish or was missing.

Note: Base multivariable model adjusts for patient race, sex, insurance, age, primary language, Elixhauser Score, and physician sex, and includes patient clinic, zip code and time fixed effects, with standard errors clustered by physician. Adjusted message content model has the same adjustments as the base model but additionally adjusts for message content. Day of year and hour of first message was used to apply time fixed effects. Adjusted clinic fixed effects model has the same adjustments as prior model but with clinic fixed effects included. Insurance defined as insurance coverage as of January 2021. Age defined as age at the beginning of 2021 based on patient's birth date. The van Walraven weighted Elixhauser score was used which calculates a weighted- score based on a patient's active comorbidities. The patient was defined as having a comorbidity condition if they had an active corresponding diagnosis code in the problem list, and the start date of diagnosis was prior to the 2021 year. *Indicates the patient did not identify their race/ethnicity among those listed.

© 2025 Rotenstein LS et al. *JAMA Network Open*.

eTable 8. Adjusted Logistic Regression Models for Proportion of Messages with Any Care Team Member Response in 1 Business Day by Patient Characteristics

	Unadjusted Analysis		Adjusted Model* with Patient Zip Code & Time Fixed Effects		Adjusted* Patient Zip Code, Time, Clinic & Dominant Topic	
	Odds Ratios (95% CI)	p-value	Odds Ratios (95% CI)	p-value	Odds Ratios (95% CI)	p-value
**Race/Ethnicity**						
White	Ref		Ref		Ref	
Asian	1.07 (1.05, 1.09)	<0.001	0.98 (0.93, 1.04)	0.55	1 (0.97, 1.03)	0.98
Black/African American	0.88 (0.86, 0.9)	<0.001	0.94 (0.9, 0.97)	<0.001	0.95 (0.91, 0.99)	0.01
Declined or Unavailable	0.93 (0.91, 0.95)	<0.001	0.94 (0.9, 0.97)	<0.001	0.96 (0.93, 0.99)	0.02
Hispanic	0.81 (0.79, 0.83)	<0.001	0.88 (0.84, 0.93)	<0.001	0.93 (0.9, 0.97)	<.001
Native American/Pacific Islander	0.92 (0.89, 0.95)	<0.001	0.87 (0.8, 0.95)	<0.001	0.89 (0.82, 0.96)	0.02
Other*	0.81 (0.76, 0.86)	<0.001	0.94 (0.9, 0.99)	0.03	0.95 (0.9, 0.99)	<0.001
**Insurance Category**						
Commercial	-		-		-	
Dual	0.92 (0.89, 0.95)	<0.001	0.79 (0.76, 0.82)	<0.001	0.8 (0.78, 0.83)	<0.001
Medicaid	0.66 (0.65, 0.68)	<0.001	0.84 (0.82, 0.87)	<0.001	0.85 (0.83, 0.88)	<0.001
Medicare	0.8 (0.78, 0.81)	<0.001	0.96 (0.94, 0.98)	<0.001	0.96 (0.94, 0.97)	<0.001
Other**	0.86 (0.85, 0.87)	<0.001	0.88 (0.77, 1.02)	0.09	0.93 (0.83, 1.05)	0.25
Unavailable	0.8 (0.74, 0.87)	<0.001	0.83 (0.74, 0.93)	<0.001	0.87 (0.78, 0.97)	0.01
**Language**						
English	-		-		-	
Spanish	0.9 (0.87, 0.93)	<0.001	0.8 (0.74, 0.86)	<0.001	1.04 (0.99, 1.08)	<0.001
Other/Missing***	0.63 (0.61, 0.66)	<0.001	0.98 (0.93, 1.03)	0.42	0.86 (0.8, 0.92)	0.08

*Indicates the patient did not identify their race/ethnicity among those listed. **Indicates that the patient had insurance listed but it was not one of the four named categories. ***Indicates that the patient's primary language was not English/Spanish or was missing.

© 2025 Rotenstein LS et al. *JAMA Network Open*.

eTable 9. Adjusted Logistic Regression Models for Proportion of Messages with Any Care Team Member Response in 3 Business Days by Patient Characteristics

	Unadjusted Analysis		Adjusted Model* with Patient Zip Code & Time Fixed Effects		Adjusted Model* with Clinic and Content FE	
	Odds Ratios (95% CI)	p-value	Odds Ratios (95% CI)	p-value	Odds Ratios (95% CI)	p-value
**Race/Ethnicity**						
White	-		-		-	
Asian	1.09 (1.07, 1.11)	<0.001	0.99 (0.95, 1.04)	0.81	1.01 (0.98, 1.03)	0.69
Black/African American	0.87 (0.85, 0.89)	<0.001	0.93 (0.89, 0.97)	<0.001	0.94 (0.9, 0.98)	<.001
Declined or Unavailable	0.93 (0.91, 0.96)	<0.001	0.94 (0.9, 0.97)	<0.001	0.97 (0.94, 1)	0.08
Hispanic	0.79 (0.77, 0.81)	<0.001	0.88 (0.84, 0.92)	<0.001	0.92 (0.89, 0.96)	<.001
Native American or Pacific Islander	0.9 (0.87, 0.94)	<0.001	0.93 (0.88, 0.98)	0.01	0.9 (0.83, 0.98)	0.01
Other*	0.81 (0.76, 0.86)	<0.001	0.88 (0.8, 0.96)	<0.001	0.94 (0.9, 0.99)	0.02
**Insurance Category**						
Commercial	-		-		-	
Dual	0.64 (0.63, 0.65)	<0.001	0.77 (0.74, 0.8)	<0.001	0.79 (0.76, 0.81)	<.001
Medicaid	0.77 (0.76, 0.78)	<0.001	0.82 (0.8, 0.85)	<0.001	0.84 (0.82, 0.86)	0.03
Medicare	0.85 (0.84, 0.86)	<0.001	0.95 (0.94, 0.97)	<0.001	0.95 (0.94, 0.97)	<.001
Other**	0.79 (0.73, 0.86)	<0.001	0.85 (0.72, 0.99)	0.03	0.87 (0.76, 0.99)	<.001
Unavailable	0.7 (0.66, 0.74)	<0.001	0.82 (0.73, 0.92)	0.00	0.81 (0.72, 0.92)	0.14
**Language**						
English	-		-		-	
Spanish	0.61 (0.58, 0.63)	<0.001	0.79 (0.73, 0.85)	<0.001	0.84 (0.78, 0.9)	<.001
Other/Missing***	0.9 (0.87, 0.93)	<0.001	0.99 (0.94, 1.04)	0.64	1.04 (0.99, 1.08)	<.001

*Indicates the patient did not identify their race/ethnicity among those listed. **Indicates that the patient had insurance listed but it was not one of the four named categories. ***Indicates that the patient's primary language was not English/Spanish or was missing.
